# Management of arthrofibrosis in neuromuscular disorders: a review

**DOI:** 10.1186/s12891-022-05677-z

**Published:** 2022-07-29

**Authors:** Edith Martinez-Lozano, Indeevar Beeram, Diana Yeritsyan, Mark W. Grinstaff, Brian D. Snyder, Ara Nazarian, Edward K. Rodriguez

**Affiliations:** 1 Musculoskeletal Translational Innovation Initiative, Carl J. Shapiro Department of Orthopaedic Surgery, Beth Israel Deaconess Medical Center and Harvard Medical School, Boston, MA 02215 USA; 2 Departments of Biomedical Engineering, Chemistry, and Medicine, Boston University, 330 Brookline Avenue, Stoneman 10, Boston, MA 02215 USA; 3 Department of Orthopaedic Surgery, Boston Children’s Hospital and Harvard Medical School, Boston, MA 02215 USA; 4 Department of Orthopaedic Surgery, Yerevan State Medical University, Yerevan, 0025 Armenia

**Keywords:** Neuromuscular, Contractures, Range of motion, Therapy, Surgery

## Abstract

Arthrofibrosis, or rigid contracture of major articular joints, is a significant morbidity of many neurodegenerative disorders. The pathogenesis depends on the mechanism and severity of the precipitating neuromuscular disorder. Most neuromuscular disorders, whether spastic or hypotonic, culminate in decreased joint range of motion. Limited range of motion precipitates a cascade of pathophysiological changes in the muscle-tendon unit, the joint capsule, and the articular cartilage. Resulting joint contractures limit functional mobility, posing both physical and psychosocial burdens to patients, economic burdens on the healthcare system, and lost productivity to society. This article reviews the pathophysiology of arthrofibrosis in the setting of neuromuscular disorders. We describe current non-surgical and surgical interventions for treating arthrofibrosis of commonly affected joints. In addition, we preview several promising modalities under development to ameliorate arthrofibrosis non-surgically and discuss limitations in the field of arthrofibrosis secondary to neuromuscular disorders.

## Introduction

Arthrofibrosis (AF), or rigid contracture of articular joints, is a common morbidity of many neuromuscular disorders (NMDs). AF manifests with appendicular weakness (hypotonia), spasticity, or both. Regardless of etiology (congenital, genetic, or acquired), injuries to the brain, spinal cord, peripheral nerves, or muscles often result in loss of active and dynamic joint motion. The decreased excursion of joints through their full range of motion (ROM), due to loss of neuromuscular motor activity and/or agonist-antagonist muscle imbalance, results in stagnant positioning of the joint over prolonged periods. Inmobilization with limited joint ROM provokes a pericapsular accumulation of fibrotic collagenous tissue and further limits mobility [1]. In addition, primary muscle pathology (i.e., fibrofatty tissue replacement) contribute to structural changes that reduce myotendinous extensibility [2].

Constricted, misaligned joints in a non-functional posture result in pain, loss of mobility, progressive muscle atrophy, osteoporosis, and diminished skin integrity. The associated disability constrains productivity and the ability for independent self-care. In addition, these sequelae contribute to psychosocial distress and increased healthcare costs (often transmitted to families and caregivers).

Current non-operative treatments for arthrofibrosis include physical therapy (PT), intra-muscular botulism toxin administration (primarily for spastic conditions), passive stretching, serial casting, and/or bracing. Surgical interventions to relieve soft-tissue contractures include tendon lengthening, aponeurotic muscle release, and capsulotomy [3]. However, many NMD patients are at high risk for complications related to abnormal scarring (with or without muscle atrophy), anesthesia, infection, and neurovascular traction injuries (i.e., avascular necrosis of femoral head) during surgical soft tissue lengthening.

We review the pathogenesis of joint contracture and arthrofibrosis as a consequence of upper (spastic) and lower (hypotonic) motor neuron syndromes, emphasizing treatment modalities and outcomes for affected joints. In addition, we emphizyse the limitations regarding diagnosis and management of arthrofibrosis secondary to neuromuscular disorders.

### Neuromuscular disorders

**Fig. 1:**
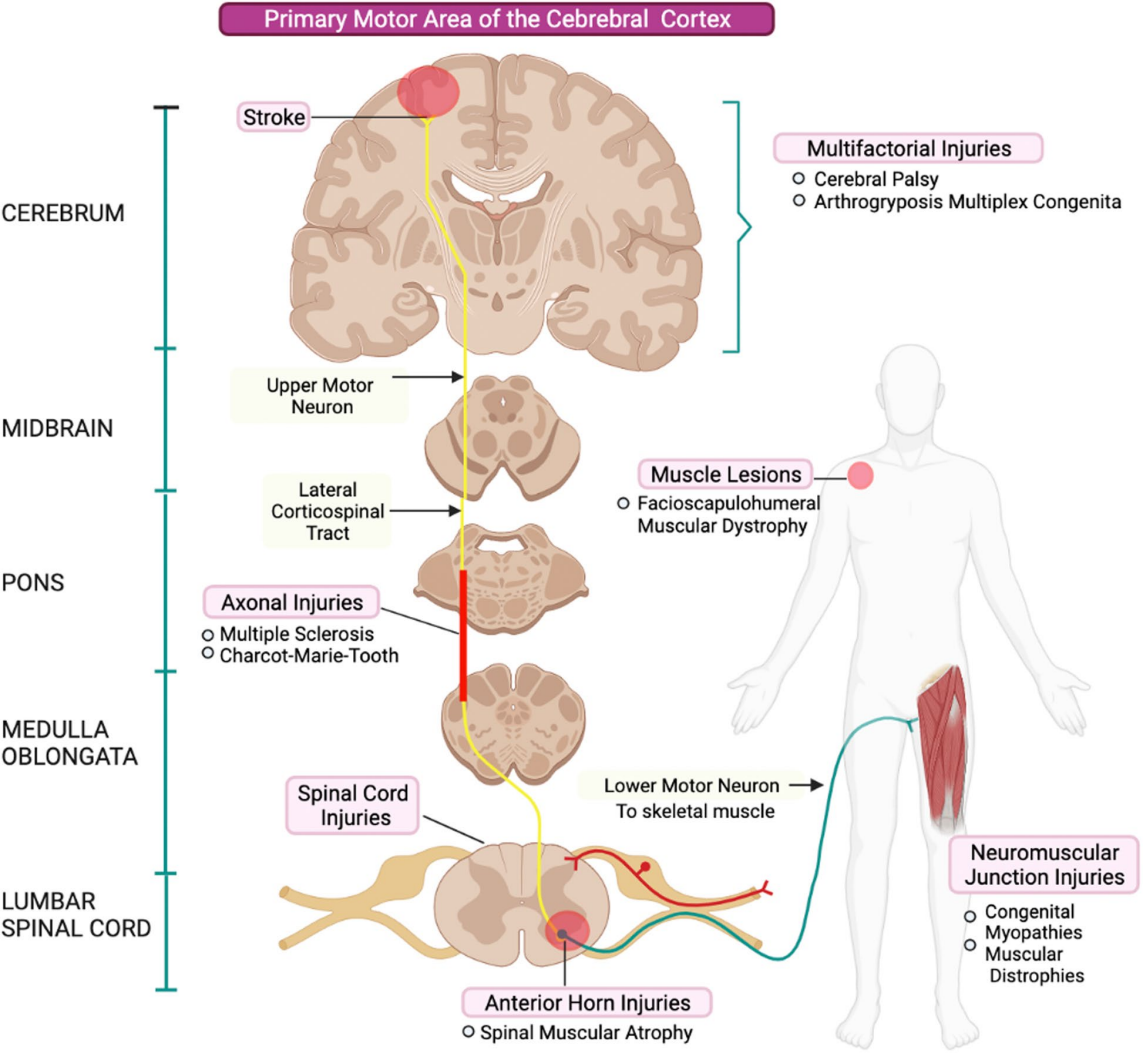
Motor Corticospinal Tract. Representation of the motor neuron pathway illustrating the anatomic site of lesion corresponding to each neuromuscular disorder [1, 4]

Neuromuscular disorders are a broadly defined group of conditions affecting the central, and/or peripheral nervous system and/or muscle. Primary and secondary NMD differentiate by their pathoanatomy and etiology (Table 1). Typically, NMDs are classified into upper (spastic) and lower (hypotonic) motor neuron syndromes (Fig. 1). Upper motor neuron (UMN) injuries to the central nervous system arise from damage to cortical motor areas or descending motor pathways in the spinal cord. Clinical manifestations are diverse and vary with the pathoanatomy of the lesion. Initially, for acquired insults such as direct trauma, ischemia (stroke, hemorrhage) or hypoxia, patients experience acute hypotonia. However, the subsequent lack of cortical inhibitory signaling increases the excitability of gamma and alpha motor neurons distally at the spinal cord.

**Table 1:**
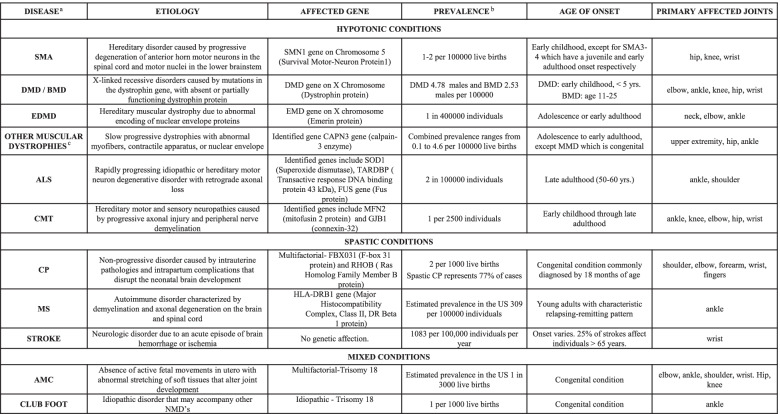
Common neuromuscular disorders - epidemiology and pathophysiology

^a^*SMA* Spinal Muscular Atrophy, *DMD* Duchenne Muscular Dystrophy, *BMD* Becker Muscular Dystrophy, *EDMD* Emery-Dreifuss Muscular Dystrophy, *ALS* Amyotrophic Lateral Sclerosis, *CMT* Charcot-Marie-Tooth, *CP* Cerebral Palsy, *MS* Multiple Sclerosis, *AMC* Arthrogryposis Multiplex Congenita

^b^Estimated worldwide prevalence except for AMC and MS

^c^Includes Limb-Girdle Muscular Dystrophy (LGMD), Myotonic Muscular Dystrophy (MMD), and Facioscapulohumeral Muscular Dystrophy (FSHD). [References] [2, 7, 25, 43, 47, 110–130]

The muscle stretch reflex increase and generates muscle spasticity and an imbalance between agonist and antagonist muscle groups crossing the joint [5]. The net result is a restriction of joint ROM and malposition.

Lower motor neuron (LMN) syndromes damage the spinal cord (distal to the conus medullaris) or peripheral axons. This causes paralysis of corresponding muscles distal to the lesion, with associated areflexia. Progressive denervation and disuse result in widespread muscular atrophy [5]. In hypotonic patients, muscle weakness and the inability to actively power joints through full ROM give rise to muscle-tendon unit contractures that restrict joint motion and diminish functional mobility [6]. Similarly, muscle dystrophies also manifest as weak, areflexic muscles. However, the deforming muscle forces and induced joint abnormalities in flaccid paralysis are not the same as in spastic paralysis. In flaccid paralysis, the myotendinous contracture is in the epimysium and fascia, with preservation of central motor control; in spastic paralysis, the muscle is shortened, collagen 1 accumulates in the endomysium and central motor control is altered [7].

**Fig. 2:**
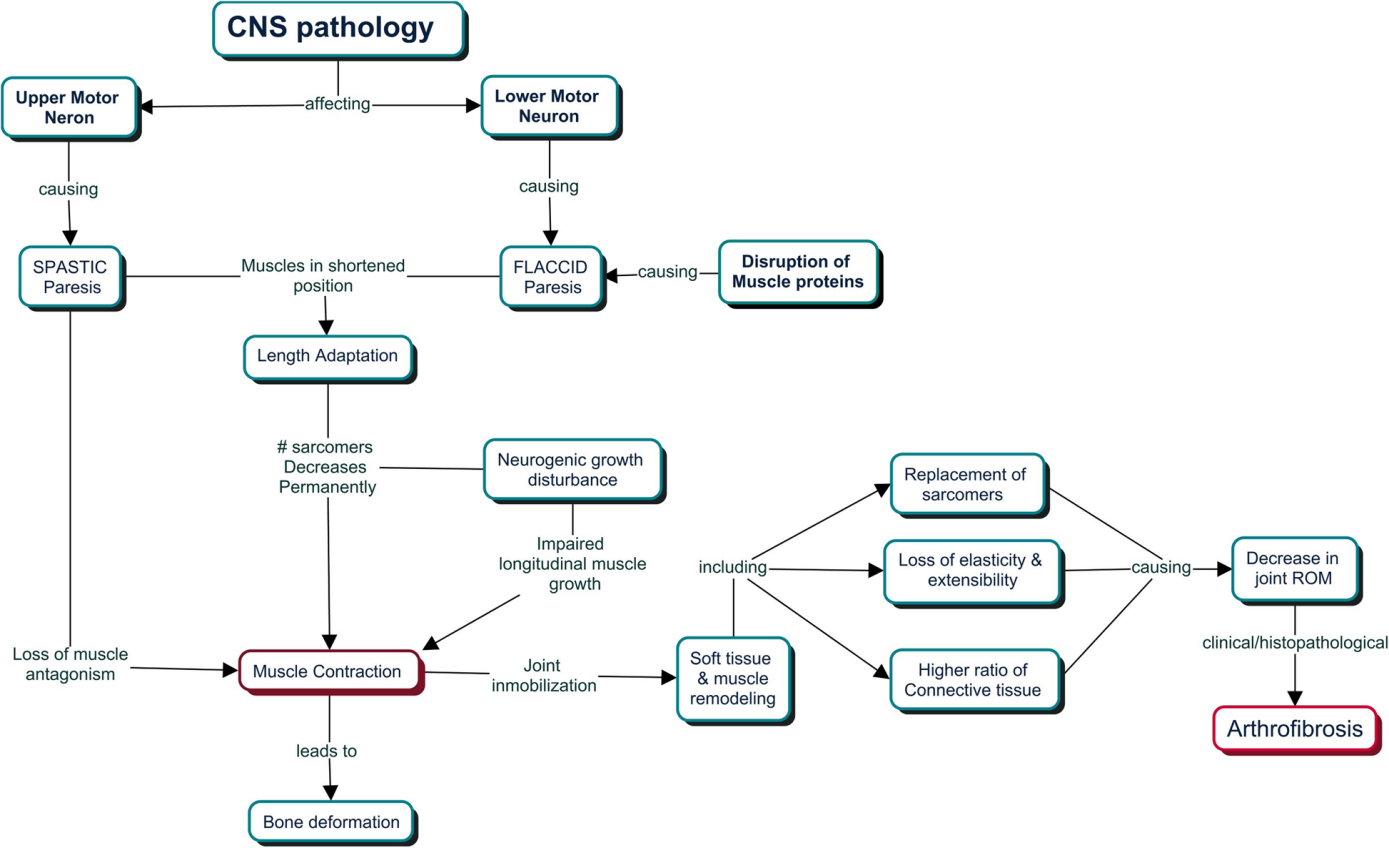
Pathophysiology of arthrofibrosis secondary to neuromuscular disorders. Central nervous system (CNS) disruptions can damage the Upper or Lower motor neuron. UMN lesions lack cortical inhibitory signaling but increase excitability in the gamma and alpha motor neurons (distally at the spinal cord), causing spastic paresis. Overactivated spastic muscles lose balance with their corresponding antagonist muscles and suffer from “pulling” the entire limb into a deformed posture. With time, the contracted (shortened) muscle undergoes length adaptation decreasing the number of sarcomeres continuously until it becomes a fixed muscle contraction. On the other hand, lesions in the spinal cord or peripheral axons represent LMN lesions. These lesions cause denervation, atrophy, and flaccid paralysis, leading to muscle contractures. Both UMN/LMN lesions may present neurogenic growth disturbances if the contractured muscle grows at a different rate than the bone (aggravating the contraction and leading to bone deformities). As the muscle shortens in patients with NMD, the joint becomes immobilized in an abnormal position. With time, pathophysiological changes in the muscle-tendon unit and the periarticular soft tissue occur, including 1) replacement of sarcomeres with fibrofatty connective tissue that will reach the joint space, 2) loss of elasticity and extensibility as the connective tissue forms more cross-bridges with collagen and 3) a higher ratio of connective to contractile tissue (as the connective tissue loss is less rapidly). All this together will ultimately cause arthrofibrosis with a decrease in ROM both histologically and clinically. [References] [2, 5, 8–12]

Regardless of the primary etiology, there are multiple biological pathways that lead to pathophysiological changes in the muscle-tendon unit and periarticular soft tissues resulting in AF (Fig. 2). Static joint posture with shortening of the muscle-tendon unit may be associated with up to a 40% loss of sarcomeres and subsequent fibrofatty replacement of functioning muscle [2]. Additionally, collagen fibers may undergo rearrangement that prevents the fibers from gliding, causing increased resistance to passive stretch and progressive joint stiffness [2].

## Patophysiology of arthrofibrosis

The exact mechanism by which multiple neuromuscular disorders culminate in AF of the major joints is not completely understood. However, the pathophysiology of AF is widely compared to the mechanism of organ fibrosis and is briefly reviewed here [8].

Arthrofibrosis has been proposed to result from an exacerbated and uncontrolled inflammatory process surrounding the joint. A chronically inflamed joint (with restricted ROM), undergoes repetitive cycles of oxidative stress and a consequently heightened inflammatory response. Several cytokines and growth factors, including interleukin (IL)-1, IL-6, IL-17, tumor necrosis factor (TNF)-α, transforming growth factor (TGF)-β, platelet-derived growth factor (PDGF), β-catenin, hypoxia-inducible factor 1α (HIF-1α) and bone morphogenic protein-2 (BMP-2) are released during these cycles [8, 9, 13, 14]. Cytokines initiate and intensify the inflammatory response recruiting important inflamatory cells including mast cells, macrophages, and lymphocytes that subsequently promote fibroblast proliferation and reduce vascularization [13].

There are many hypothesized pathways that lead to arthrofibrosis. A detailed discussion of these pathways is beyond the scope of this review. However, we explore 3 major determinants of fibrosis that have been linked to arthrofibrosis secondary to neuromuscular disorders.

1. *Cytokines*

Pro-fibrotic cytokines are thought to cause an imbalance between ECM production and degradation, leading to excessive deposition of matrix proteins [13]. One of the most recognized cytokine is TGF-β, a ubiquitous signaling protein with prominent roles in tissue repair and scar formation [8, 15]. TGF-B, along with ß-catenin (activates fibroblast via the WNT pathway), leads to the activation and differentiation of myofibroblasts which produce *COL1A1* and *COL1A2*, creating a higher ratio of collagen type 1 to elastin (the stretchy component of a healthy ECM) [13].

Recent literature has found that upon muscle injury, seen in Duchenne Muscular Dystrophy as well as other inherited and acquired myopathies, TGF-β is strongly activated. This activation elicits a downstream SMAD signaling to repair the injury and restore muscle function [16]. However, the dystrophic muscles in these patients are characterized by excessively elevated TGF-β activity, which exacerbates the inflammatory response and aggravates the fibrotic process [17].

2. *Hypoxia*

Fibrotic tissue resulting from AF is characterized by reduced vascularity and a state of chronic hypoxia. It has been proposed that the continue lack of oxygen in the ECM lowers the pH, increases levels of lactic acid and activates TGF-B and myofibroblasts. In vivo experiments, under these circumstances, have shown that muscle regeneration is delayed and that hypoxia itself can exacerbate the expression of ECM proteins in epidermal fibroblasts in patients with systemic sclerosis [18]. In addition, there is evidence showing that altered skeletal muscle regeneration cycles (under hypoxia), seen in muscular dystrophies, result in asynchronous remodeling of the microenvironment and subsequent fibrosis [19].

Many molecules have been implicated in the hypoxic pathway, however, the main Hypoxia Inducible Factor (HIF) responsible for inducing ECM fibrosis is Hypoxia-Inducible Factor-1α (HIF-1 α). Evidence points out that HIF-1α, found within fibroblasts and myoblasts of contractured joints of Emery-Dreifuss muscular dystrophy patients, can upregulate connective tissue growth factors and genes involved in ECM deposition, further emphasizing the importance of hypoxia in the mechanism of AF [8, 15, 20, 21].

3. *Abnormal regulation*

Fibrosis of the ECM results in a dense fibrous tissue with extensive cross-linking that becomes difficult to degrade. Typically, inflammatory cytokines are downregulated after a period of time. However, AF is believed to arise from repeated trauma and long-term inflammation in the joint. This results in continued activation of myofibroblasts with non-existent resolution [8, 15, 20, 21]. In addition, failure of autophagy and a lack of apoptosis within the fibrotic tissue result in excess fibrosis within periarticular tissues [22].

Multiple studies are investigating AF as a consequence of fibroblast’s resistance to apoptosis. Zanotti et al. found that muscle-derived DMD fibroblasts, due to their primary genetic defect (dystrophin absence), can be characterized as pro-fibrotic. These fibroblasts are more adhesive, have a greater tendency to migrate, and are more resistant to apoptosis, than fibroblasts present in the normal population [23]. Moreover, De palma et al. state that, in Duchenne Muscular Dystrophy, the treatment of abnormal autophagy pathways such as AKT (Protein Kinase B) and mTOR (mammalian target of rapamycin) results in significantly reduced muscle inflammation and fibrosis questioning whether this pathway may also be an important pharmacological target [24].

### Principles of managing joint contractures in NMD

**Fig. 3:**
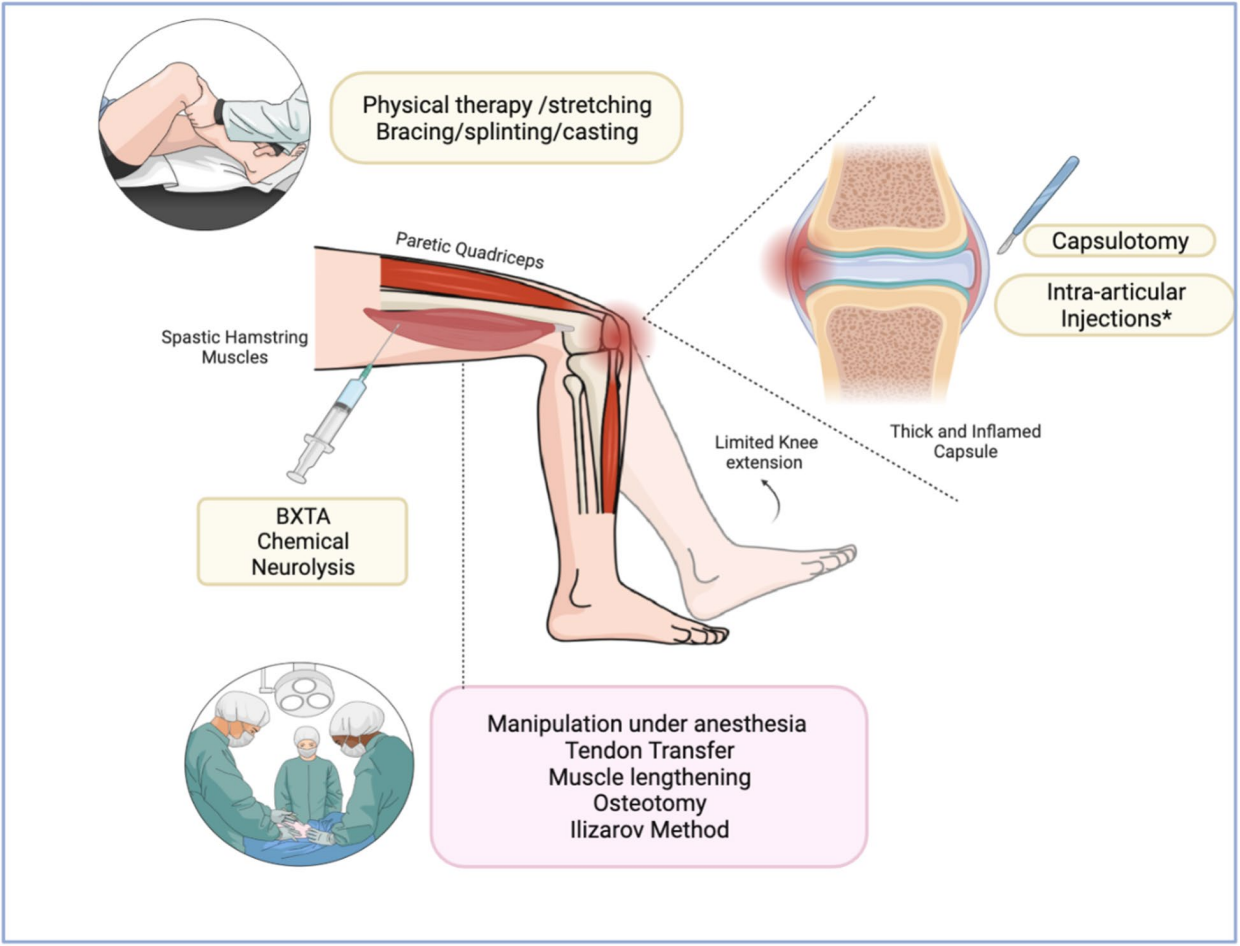
Representation of knee arthrofibrosis and available treatment therapies. *Intra-articular injections include corticosteroids and antifibrotic treatments such as novel relaxin-2 and collagenase

The treatment goal of AF is to prevent bone and joint deformity by balancing muscle forces about the joint (Fig. 3). Multiple strategies exist to achieve this goal. For example, non-operative treatments aim to improve joint mobility by performing physical therapy. Exercising and stretching the muscle may 1) delay the onset of contractures, 2) combat the deconditioning that occurs with immobility, and 3) alleviate contractures through passive and active-assisted stretching [25]. Sodhi et al., show a constant self-reported improvement in mobility in 90% of their population (patients with no NMD but elbow, forearm, wrist and/or knee fibrosis) after 10 years of a consistent daily stretching program, augmented by static and/or dynamic orthosis [26]. In NMDs, however, improvements in functional ROM are usually transient and long-term effectiveness is limited and unpredictable [27]. Moreover, there are few randomized clinical trials (RCTs) in patients with NMDs, and commonly the initiation and duration of physical therapy remain a decision of the clinician.

For spastic muscles, intramuscular chemodenervation using Botulinum toxin A (BTXA) may be used. BTXA inhibits acetylcholine release at the presynaptic neuromuscular junction and therefore helps ameliorate muscle spasticity. Randomized placebo control studies demonstrate a temporary (3–6 months) relief from spasticity and improved joint ROM [28–30]. Alternatively, alone or in synergy with BTXA, dilute ethanol or phenol (carboxylic acid) can be used to selectively demyelinate peripheral nerve motor branches from specific muscle groups [31]. The resulting blockade slows the efferent signal for spastic muscle activation, thereby decreasing the intensity of the contraction. In addition, selective weakening of targeted spastic muscles increases the efficacy of non-operative treatments by 1) facilitating the passive stretching of shortened agonist muscles, 2) enhancing the active strengthening of antagonistic muscles, and 3) improving selective motor control through the administration of directed electrical stimulation [32, 33]. Graham et al. emphasize the importance of physiotherapy and the use of orthoses or serial cast application to re-align deformed joints to maximize the benefits of myoneural blockade [34].

It is important to recognize that myoneural blockade is not for hypotonic neuromuscular pathologies, as it further weakens already weakened muscles. In addition, the selection of patients undergoing neurolysis requires a thorough analysis, as neurolysis may induce fibrosis in all tissue layers of the nerve. Until recently, there has been little interest in the role that nerves play in initiating or maintaining arthrofibrosis. Yoon and Kim (2018) have shown that in the kidney, afferent nerve-derived Calcitonin Gene-Related Peptide (CGRP) upregulates TGF-B and Connective Tissue Growth Factor (CTGF) to induce fibrosis [35]. TGF-B additionally promotes the expression of an ECM remodeling factor (augmented in DMD) protein Cellular Communication Network factor 2 (CCN2). According to Gallardo et al., overexpression of this protein causes increased expression of collagens, fibronectin, and myofibroblast α-smooth muscle actin (α-SMA), suggesting an expansion of the myofibroblast population [36]. These findings suggest that nerve damage, from any etiology, is likely responsible for releasing multiple profibrogenic factors that could be implicated in arthrofibrosis. Thus the use of neurolysis and its potential role in arthrofibrosis requires further evaluation in validated animal models reproducing neuromuscular disorders.

Similarly, clinicians need to be aware that although injections are remarkably low risk and are routinely used in a non-surgical setting, the risk of pain, swelling, bruising, infection, and local diffusion of botulinum toxin in adjacent areas has been reported. To minimize risks, the joint can be accessed safely with ultrasound or fluoroscopy if needed. Stretch-induced neuropathy is unrelated to injection but rather associated with ambitious manipulation of a stiff limb in the context of established joint fibrosis.

Soft tissue release and myotendinous lengthening may be indicated when non-surgical treatments fail to mitigate progressive joint contractures that restrict functional mobility, cause pain and contribute to skin breakdown. Anatomically where the muscle-tendon unit is elongated, determines the magnitude of length achieved. Intramuscular aponeurotic recession achieves a minimum change in myotendinous length. In contrast, tendon lengthening results in a more significant myotendinous length but higher muscle weakness. After tendon lengthening, the tendon is longer, but the muscle fibers (sarcomeres) shorten [37]. The effect of surgical lengthening on muscle force can be inferred from the parabolic length-tension relationship for skeletal muscle (Blix curve). The maximum isometric tension is generated at the muscle’s resting length (apex of the parabola), where the number of cross-bridges formed between the myosin and actin filaments is maximum [38]. Relative to this resting length, a shortened muscle, either by contracture or as a consequence of tendon lengthening, is a weaker muscle, as is an overlengthened muscle. To reestablish the proper length-tension function relationship, lengthening approximately 50% of the affected shortened muscle helps restore its “normal” resting length, where ~ 0.5 mm of musculotendinous lengthening is required for every degree of joint contracture [39]. In general, myotendinous lengthening will not improve the work that a muscle can perform, (i.e., integral of Blix curve). The offset engendered by the elongated muscle-tendon unit simply shifts the Blix curve to the right, allowing the joint to assume a more functional position. Thus, for spastic conditions, myotendinous lengthening not only increases the functional excursion of the muscle-tendon unit, but since the lengthened muscles contract less efficiently, it diminishes the deforming force of the spastic agonist muscles. As a result, muscle lengthening affords a more balanced action of the previously overstretched antagonist muscles [40]. In contrast, intramuscular aponeurotic recession is preferred for hypotonic conditions, with myotendinous contracture residing in the epimysium and fascia. The patient receives a peri-capsular release, if the joint itself remains rigidly contracted, after myotendinous lengthening. Unstable, dysplastic joints require open reduction and likely redirectional osteotomies or resection arthroplasty.

### Managing upper and lower extremity contractures

Although there is insufficient evidence supporting different interventions specifically to improve joint ROM in NMDs there are some accepted treatment modalities to minimize the impact or disability from the contractures seen in these patients [2, 8, 41]. The following section will highlight the most common treatment modalities applied for upper and lower body contractures seen in patients with NMDs.

### Upper extremity contractures in NMD

Although there is a high prevalence of upper limb involvement in NMDs, treatment of these contractures is approached less frequently than in lower limb deformities, since these contractures do not directly affect standing posture, balance, or gait. Upper limb abnormalities can impose a significant burden on quality of life when shoulder, elbow, wrist and finger contractures interfere with the performance of activities of daily living such as feeding, toileting, hygiene, and dressing. The typical posture assumed is adduction of the shoulder, flexed elbow, pronated forearm, flexed and ulnar deviated wrist, and an adducted thumb with finger flexion contractures [2, 42]. Overall, intervention is indicated when contractures interfere with function, affect personal hygiene, hinder positioning, or cause intertrigo [43] .

#### Management of shoulder contracture

Adduction and internal rotation shoulder contractures result from muscular imbalance around the glenohumeral joint and contribute to difficulty with overhead motion and axillary hygiene. Shoulder contractures present with spastic shortening of the pectoralis major, subscapularis, and latissimus dorsi muscles [40, 43]. Passive stretching with or without BTXA, electrical stimulation, heat, and muscle massage may improve glenohumeral ROM [33, 44, 45]. Robot-assisted physiotherapy has been reported to improve shoulder motion in stroke patients after 3 months [46].

Recalcitrant contractures require step-cut-Z lengthening or complete tendon release of the subscapularis and pectoralis major muscles. Biceps brachii lengthening and partial joint capsulotomy can also be performed if necessary [43, 47]. Overlengthening is associated with loss of internal rotation power and with external rotation contractures, thus a thorough analysis of the deforming muscle forces must be conducted before surgery [48].

#### Management of elbow contracture

Elbow flexion contractures are ubiquitous in both hypotonic and hypertonic NMDs, typically ~ 35° [49]. Physiotherapy attempts to stretch the biceps brachii to increase extension ROM. Resistant elbow deformity is treated with BTXA to the biceps, brachialis and brachioradialis followed by sustained mechanical stretching using serially applied long arm extension cast or dynamic hinged elbow extension braces [50]. Dynamic bracing may exacerbate antagonist muscle tone in dystonia [50, 51]. Vibratory and electric stimulation of the antagonist tricep muscles is also utilized in combination with physical therapy to counteract spasticity of the biceps brachii, increase extension and gain ROM [52] . Myotendinous surgical release is indicated for elbow flexion contractures greater than or equal to 60° that cause skin breakdown in the cubital fossa and interfere with activities of daily living [53]. This procedure can involve complete or partial lengthening of the biceps brachii tendon, partial release of the proximal brachioradialis muscle, lacertus fibrosus release and anterior elbow capsulotomy [54, 55]. Lengthening the biceps can result in diminished active forearm supination and elbow flexion. When manioulating the elbow, the median, ulnar and radial nerves are at risk for stretch neuropraxia [55, 56]. In addition, heterotopic bone formation is seen in patients with acquired (ischemic, traumatic) brain and spinal cord injuries who develop severe upper extremity spasticity [57]. The prescence of heterotopic bone can compliacate surgical planning, increase bleeding, and the risk of neurovascular injury if the heterotopic bone involves these structures. Hence, lengthening surgery should be individualized and planned carefully.

#### Management of wrist and hand contracture

Wrist and hand involvement that interferes with grasp can occur in children or adults afflicted with spastic or paralytic conditions. Spastic hands must be differentiated from those with dystonic posturing. During sleep or sedation, the dystonic hand does not exhibit hyperreflexia or fixed myotendinous contractures [39]. Flexion with concomitant ulnar deviation is the most common wrist deformity, provoked by the unbalanced activity of the flexor carpi ulnaris muscle (FCU) [2]. Wrist flexion deformity also contributes to the musculotendinous shortening of the extrinsic (deep and superficial) finger flexors. Stretching with passive and dynamic splinting of wrist and finger flexors are the mainstays of a rigorous physiotherapy program. Neuromuscular blockade with BXTA to the FCU and/or extrinsic finger flexors effectively reduces spasticity, increases ROM, and improves functional hand movement [58]. Non-invasive strategies can also be used to rehabilitate motor and sensory neural pathways. A systematic review by Inguaggiato et al. shows neuroplastic changes in the sensorimotor cortex (i.e. enlargement of the primary hand motor area contralateral to the paretic hand) of children with unilateral cerebral palsy [59]. These changes are associated with improvement in hand motor function after constraint-induced movement therapy (CIMT), virtual reality therapy, or bimanual training programs [59, 60]. When conservative treatments do not improve range of motion, surgical interventions are indicated. Myotendinous lengthening of the FCU is required for wrist flexion contractures greater than or equal to 50°. For excessive fixed forearm pronation, lengthening the pronator teres is appropriate, keeping in mind that most daily activities are performed with the forearm in pronation. For rigid finger flexion deformities involving the MTPJ and/or DIPJ, lengthening the flexor digitorum superficialis, the flexor digitorum profundus at the muscle aponeurosis (moderate deformity) or multiple tendons at the forearm (severe deformity) is necessary. In the setting of moderate spastic contracture of the FCU with concomitant weakness of the wrist extensors, transfer of the FCU tendon to the extensor carpi radialis longus (ECRL) or brevis (ECRB) to augment the weak antagonist wrist extensors is effective in improving wrist posture and function [61, 62]. However, extension deformities are reported as a complication of muscle-tendon transfers performed on individuals who have not reached skeletal maturity [63]. Use of external fixators like the Ilizarov apparatus can correct rigid wrist flexion contractures > 60°. For severe palmar flexion deformity, one can perform a combination of soft tissue releases and bony procedures (distal radius osteotomy, proximal row carpectomy, wrist fusion) to restore neutral wrist posture [64]. Nevertheless, these surgeries are not curative of the primary disease and many patients will experience meaningful contracture recurrence requiring further management.

### Lower extremity contractures in NMD

Lower extremity contractures affect standing posture, balance and functional mobility required for ambulation and the performance of activities of daily living such as hygiene, toileting and dressing; thus, early and aggressive treatment is indicated. Lower extremity contractures rarely occur in isolation, muscles that cross two joints are most frequently affected; proximal contractures affecting the hip and knee are as common as the equinus position of the foot, which is the most visible of the abnormal lower extremity postures. Fixed versus dynamic contractures should be treated differently (as fixed contractures significantly impact function show minimum response to conservative interventions [2]. Simultaneous, multi-level interventions that address all co-existent deformities about the hip, knee and ankle may improve posture and gait better than multiple isolated procedures performed at different anatomic sites serially over time [65].

#### Management of hip contractures

Debilitating flexion-adduction contractures about the hip occur in spastic and paralytic conditions as a result of the lack of erect weight-bearing posture and muscle imbalance. Agonist hip adductors and flexors (iliopsoas) show greater contracture force against antagonist abductors (gluteus medius, tensor fascia lata) and extensors (gluteus maximus, hamstrings) [66, 67]. Chronic flexion and adduction of the hip contribute to posterolateral migration of the femoral head with subsequent hip subluxation [66]. Anterior branch obturator nerve block (phenol, alcohol) with or without BTXA to adductors and iliopsoas combined with structured physiotherapy (static and dynamic stretching) and hip abduction bracing improves hip ROM and compensatory gait mechanisms (anterior pelvic tilt, contralateral step length) in ambulant children, but only temporizes the condition in non-ambulant children [68–71]. Soft tissue release remains the mainstay of treatment for fixed contractures about the hip facilitating positioning, perineal care (toileting, hygiene) and standing posture [72]. Tenotomies of the adductor longus and brevis, release of the gracilis, lengthening of the psoas tendon (at the pelvic brim for ambulatory patients) or iliopsoas tendon (at the lesser trochanter for non-ambulatory patients) can alleviate the deforming forces and partially correct muscular imbalance from weak gluteals [73, 74]. For spastic patients, these procedures are often combined with BTXA and chemical neurectomy of the anterior branch of the obturator nerve [75]. Post-operative bracing and structured physiotherapy to maintain hip abduction and extension are mandatory for 3–4 months. However, soft tissue procedures alone are unlikely to mitigate progressive hip instability, with re-subluxation rates as high as 77% [76]. Complications include wound infections, heterotopic ossification, injury to the femoral neurovascular bundle, and avascular necrosis of the femoral head [77, 78].

#### Management of knee contractures

Knee flexion contractures develop as a consequence of muscle imbalance between the quadriceps (knee extension) and hamstrings (knee flexion). In erect posture, the hamstrings are a strong hip extensor and weak knee flexor (short head biceps femoris), however in crouched posture provoked by contracted psoas and hamstrings, there is positional lever arm dysfunction: the hip lever shortens and knee lever lengthens, such that the hamstring becomes a better knee flexor and a weaker hip extensor [79]. Physiotherapy consisting of a structured program of gait training, standing, passive hamstring stretches and static or dynamic extension splinting of the knee performed daily may be effective, but is typically combined with BTXA injections into the hamstrings and chemical neurolysis of medial hamstring motor branches as an adjuvant to physical therapy in spastic conditions, to decrease flexor agonist muscle tone [2, 31, 80–84].

Moderately severe knee flexion deformities (flexion > 20° in stance phase of gait cycle, popliteal angle > 60°, sacral sitting) refractory to conservative treatments are traditionally managed by fractional hamstring lengthening (tenotomy of semitendinosus, gracilis and aponeurotic recession of semi-membranosis) with or without myoneural blockade [85–87]. Baumann et al. show improvement in passive knee extension (restriction from 25° before surgery to 5° at average 32 months follow-up) after fractional hamstring lengthening in patients with spastic CP (*n* = 17) [88]. However, hamstring lengthening performed in isolation, when there are co-existing abnormalities at the hip and ankle may result in unfavorable outcomes such as genu recurvatum because of a shortened gastrocnemius and/or hyperlordosis reflecting a shortened iliopsoas and impairment of the hamstring’s hip extensor function. Knee flexion contractures may recur after hamstring lengthening in children as they grow. Severe rigid knee flexion contractures (> 20°) demand a combination of soft tissue releases, supracondylar extension femoral osteotomy (shortening the femur minimizes risk of sciatic stretch neuropathy from manipulation), and patellar tendon advancement to re-tension the quadriceps and rebalance the knee extensor mechanism [67, 89].

#### Management of ankle contractures

Plantarflexion contracture of the ankle (equinus) is a frequent complication of spastic and paralytic NMDs (93% prevalence of equinus foot in spastic CP) [90] . If implemented early and consistently, active and passive stretching of the gastroc-soleus complex significantly improves ankle dorsiflexion range [91]. Serial casting and/or use of orthotic devices in conjunction with a therapy program can enhance the efficacy of stretching exercises [92, 93]. Ankle foot orthotics ameliorate plantarflexion deformity and can eliminate foot drop during the swing phase of the gait cycle. However, stretching alone does not provide sustained long term improvements [94–96]. To augment passive and dynamic stretching of the shortened calf muscles, BTXA administered to the spastic gastroc-soleus complex (agonist) in combination with strengthening of the tibialis anterior muscle (antagonist) can enhance ankle dorsiflexion motion [94, 97–99].

Surgical lengthening of the constricted gastroc-soleus myotendinous complex is appropriate for patients unresponsive to non-operative treatments [100, 101]. Recession of the gastrocnemius aponeurosis alone, or differentially in combination with recession of soleus aponeurosis, is preferred to alleviate equinus deformity and to avoid “over-lengthening” manifest as excessive ankle dorsiflexion and crouched posture (especially in the context of concomitant hamstring contracture). Achilles tendon lengthening, either as an open Z-lengthening or percutaneous tendon slide is very effective in relieving ankle equinus [92, 93], but at the increased risk for excessively lenghening/weakening the gastroc-soleus muscle-tendon unit. In paralytic conditions, the stiffness of the constricted gastroc-soleus complex can partially substitute for underlying muscle weakness. Complications include sural nerve injuries, infections, scarring, secondary fibrosis, and muscle atrophy [99, 102].

## Future directions

Pharmacological treatments targeting the primary fibrotic activators (TNF-α, IL-1, TGF-β and HIF-1α) are promising options for managing joint contractures as these inflammatory cytokines are believed to be responsible for initiating and sustaining the arthrofibrotic cascade [8, 15, 20, 21].

Collagenase injections are approved by the Food and Drug Administration (FDA) to treat chronic fibrotic tissue disorders such as Dupuytren’s and Peyronie’s disease. Collagenase, a proteolytic enzyme from *Clostridium histolyticum* breaks down peptide bonds in collagen. Villegas et al. report a significant reduction of dermal fibrosis in a scleroderma mouse model treated with a novel polymeric nanocapsule containing collagenase [103]. The controlled and sustained release of collagenase from the nanocapsules over time improves outcomes [103]. However, collagenase has not been studied as a therapeutic intervention for joint contractures secondary to NMDs.

Halofuginone, a quinazolinone alkaloid isolated from the plant *Dichroa febrifuga*, was approved by the FDA as an inhibitor of collagen I synthesis. Multiple studies report that halofuginone reverses fibrosis in various animal models and human diseases [104]. Halofuginone is used clinically to treat scleroderma. After 6 months of topical application, a marked reduction in collagen synthesis occurred in the skin of a patient with cutaneous graft versus host disease, a condition marked by significant skin fibrosis and contractures [105]. Further research is required to evaluate its potential efficacy on NMD-related joint contractures.

Relaxin-2, an antifibrotic peptide hormone secreted prior to childbirth, inhibits fibrogenesis and collagen overexpression. Multiple intra-articular injections of recombinant relaxin-2 ameliorated ROM deficits in a rat model of shoulder arthrofibrosis as demonstrated by biomechanical measurements and histological findings [106]. Such developments show promise for addressing non-surgical arthrofibrotic contractures observed in NMDs.

It is noteworthy to emphasize that the current understanding of arthrofibrosis is limited as the cellular heterogeneity of the joint (muscle, bone, synovial, and immune cells) impedes the establishment of in vitro models that successfully replicate arthrofibrosis [41]. However, fibrosis and arthrofibrosis, as previously stated, are hypothesized to share similar pathways presenting 1) increased pro-inflammatory cytokines, 2) a chronic hypoxic state, and 3) decreased apoptotic pathways. Future therapies, to not only treat fibrosis but also reverse the established fibrosis thus depend on the understanding and targeting of these and other pathophysiological pathways.

## Limitations of current literature

Despite arthrofibrosis occurring across a wide spectrum of NMD, reports are rare pertaining to arthrofibrosis as a consequence of inmobilization resulting from UMN or LMN disorders. A root problem may be the lack of standardized, accepted criteria for the diagnosis, classification and grading of arthrofibrosis [107]. Even something as basic as terminology is inconsistent as arthrofibrosis is commonly referred to in the clinical literature by various terms such as joint contracture, frozen shoulder/other joint, adhesive capsulitis, and stiff knee/ other joint [8]. Some clinicians consider arthrofibrosis a clinical finding characterized by limited ROM, while others define it in terms of histological findings of increased collagenous fibrotic deposits and adhesions in the articular or periarticular tissues.

As joint immobilization develops in patients with NMD, ROM decreases. However, reliable methods to assess ROM of arthrofibrotic joints are not standardized [108]. Measurements can include peak flexion, peak extension, overall range, or both. Furthermore, motion is often passive, or active, or against resistance, and in many studies this information is lacking or not specified. Clinically, measurements can be roughly assessed with a simple protractor in a weight or non-weight bearing position. More sophisticated techniques involve the use of digital devices and radiofrequency or reflective markers for motion tracking. At present, there is no standardized clinical method to measure joint ROM in the clinical setting.

Clinicians treat arthrofibrosis from NMD, via conservative and non-conservative treatments. The treatment decision is made after careful examination of the affected joint and after balancing improvement versus possible recurrence of contracture. There are numerous types of interventions (bracing/casting, BOTXA application, surgeries) that can be performed to relieve AF. Unfortunately, there is limited evidence pertaining management of AF secondary to NMD to evaluate their success/failure rates. The main problem when evaluating a specific intervention in NMD is a lack of RCT’s with the literature mostly consisting of case-controls, case reports, and case series with a small sample size (as there are obvious ethical dilemmas in withdrawing the current standard of treatment in order to perform controlled studies). In addition, most patients with NMD undergo multiple treatment interventions, making it difficult to isolate results from specific therapies.

Current evidence suggests that non-operative treatments (exercise, stretching, bracing/casting) may improve muscle function in patients with NMDs. However, the appropriate frequency, intensity, and duration of exercises/stretching/casting/bracing are still unclear. Different protocols have been used without establishing definite conclusions, suggesting that non-conservative treatments for one disorder might not be fully applicable for other NMDs [25].

Similarly, operative treatments are sometimes indicated for treating relapsing or resistant AF. Although there is limited evidence of secondary fibrosis after surgery in neuromuscular disorders, there is extensive literature of surgery-induced fibrosis in patients without neuromuscular disorders. This can be explained by the increased inflammatory response expected after manipulation. The pathophysiology process is under review, as there is no current method to determine how individual patients will respond. Potential new therapies for treating arthrofibrosis secondary to surgery have been suggested including the use of omega 3, capsaicin, low sugar intake, soy products, collagenase, TGF-β antibodies, IL-1 antibodies, and TNF-α [8]. These therapies may reduce, suppresses, or even reverse the inflammatory response and decrease the differentiation of myofibroblasts which could prevent or treat joint fibrosis with typically fewer risks [109]. However, these findings cannot be extrapolated to all patients and should be addressed specifically for neuromuscular disorders.

Ultimately, many surgeries require casting/bracing after surgical procedures. However, there is an absence of guidelines for casting/bracing following surgery despite prolongued immbolization is a well known contributing factor for AF [2, 8]. Currently, it is a clinical decision to determine how long a period of immobilization is needed to secure early stability but not result in significant ROM loss, particularly in adults. Protocols for cast/brace immobilization are broad, and different for diverse conditions and patient ages. There is no set rule or standardized protocol and therefore a successful immobilization protocol depends widely on the expertise of the clinician.

## Conclusions

Joint contractures resulting from UMN and LMN lesions are a debilitating consequence of multiple NMDs associated with significant morbidity, psychosocial burden, and financial cost to affected individuals and their families. While the pathophysiology remains unclear, there is consensus that joint contractures arise from adaptive soft tissue changes. Spastic and/or flaccid paralysis restrict active joint motion and manifest with chronic, static joint positioning that progress to arthrofibrotic contractures. Treatment aims to maintain functional mobility by balancing agonist-antagonist muscle forces across the joint to prevent bone and joint deformity. Current treatments focus on physical therapy, serial splinting and orthoses to stretch shortened muscle-tendon units and peri-capsular contractures. Neuromuscular blockade is an effective adjunct to augment physiotherapy in spastic conditions by relaxing overly-active agonist muscles. For recalcitrant deformities, surgical procedures such as aponeurotic muscle release, tendon lengthening, and capsular release are appropriate for increasing the functional excursion of the muscle-tendon unit. The lengthened muscles contract less efficiently, thereby diminishing the deforming force of the spastic agonist muscles, and allowing for more balanced action of the (previously overstretched) antagonist muscle. Unstable and/or dysplastic joints require bony procedures in addition to soft tissue releases.

In summary, joint contractures resulting from UMN and LMN lesions are common and severely impact quality of life. Significant opportunities exist to improve patient care by implementing novel targeted pharmacological interventions or by developing newer surgical procedures. However, without 1) proper definition and understanding of the pathophysiology of arthrofibrosis secondary to NMD, 2) no consensus in ROM assessment, 3) no concise validated outcome measures to predict the prognosis or the effect that treatments should have, and 4) no validated protocols for non-conservative therapies and casting/bracing pos surgery there will continue to be a significant gap pertaining arthrofibrosis as a consequence of NMDs. Extensive research is needed to elucidate the pathophysiology of AF and the appopriate management protocols as readers currently face high variability and individual interpretations in the literature pertaining to this topic.

## Abbreviations

α-SMA: α-Smooth Muscle Actin
AF: Arthrofibrosis
ALS: Amyotrophic Lateral Sclerosis
AMC: Arthrogryposis Multiplex Congenita
BMP: Bone morphogenic protein
BMD: Becker muscular dystrophy
BTXA: Botulinum toxin
CCN2: Celullar Communication Network factor 2
CDM: Congenital myotonic dystrophy
CMT: Charcot-Marie-Tooth
CGRP: Calcitonin Gene-Related Peptide
CIMT: Constraint-induced movement therapy
CP: Cerebral Palsy
CTGF: Connective Tissue Growth Factor
DMD: Duchenne muscular dystrophy
ECM: Extracellular matrix
ECRB: Extensor carpi radialis brevis
ECRL: Extensor carpi radialis longus
EDMD: Emery-Dreifuss muscular dystrophy
FCU: Flexor carpi ulnaris
FDA: Federal Drug Administration
FSHD: Facioscapulohumeral Muscular Dystrophy
LGMD: Limb-Girdle muscular dystrophy
LMN: Lower motor neuron
MMD: Myotonic Muscular Dystrophy
MS: Multiple Sclerosis
MTPJ: Metatarsophalangeal joint
NMD: Neuromuscular Disorder
PDGF: Platelet derived growth factor
PIPJ: Proximal interphalangeal joint
PT: Physical therapy
ROM: Range of motion
SMA: Spinal Muscular Atrophy
TGF: Transforming growth factor
TNF: Tumor necrosis factor
UMN: Upper motor neuron
